# Effect of Metal Nanopowders on the Performance of Solid Rocket Propellants: A Review

**DOI:** 10.3390/nano11102749

**Published:** 2021-10-17

**Authors:** Weiqiang Pang, Yang Li, Luigi T. DeLuca, Daolun Liang, Zhao Qin, Xiaogang Liu, Huixiang Xu, Xuezhong Fan

**Affiliations:** 1 The Third Department, Xi’an Modern Chemistry Research Institute, Xi’an 710065, China; yang12110420@163.com (Y.L.); liuxg88066@163.com (X.L.); xhx204@163.com (H.X.); xuezhongfan@126.com (X.F.); 2 Science and Technology on Combustion and Explosion Laboratory, Xi’an Modern Chemistry Research Institute, Xi’an 710065, China; qzhao087@hotmail.com; 3 Space Propulsion Laboratory (SPLab), Politecnico di Milano, I-20156 Milan, Italy; luigi.t.deluca@gmail.com; 4 Key Laboratory of Energy Thermal Conversion and Control of Ministry of Education, School of Energy and Environment, Southeast University, Nanjing 210096, China; ldl@seu.edu.cn

**Keywords:** material chemistry, solid propellant, nano-sized metal particles, hazardous properties, combustion performance

## Abstract

The effects of different types of nano-sized metal particles, such as aluminum (nAl), zirconium (nZr), titanium (nTi), and nickel (nNi), on the properties of a variety of solid rocket propellants (composite, fuel-rich, and composite modified double base (CMDB)) were analyzed and compared with those of propellants loaded with micro-sized Al (mAl) powder. Emphasis was placed on the investigation of burning rate, pressure exponent (*n*), and hazardous properties, which control whether a propellant can be adopted in solid rocket motors. It was found that nano-sized additives can affect the combustion behavior and increase the burning rate of propellants. Compared with the corresponding micro-sized ones, the nano-sized particles promote higher impact sensitivity and friction sensitivity. In this paper, 101 references are enclosed.

## 1. Introduction

High-energy solid propellants play an essential role in propulsion for space exploration [1,2]. The specific impulse or density specific impulse of propellants can be increased by the inclusion of certain reactive metal powders, such as aluminum (Al), magnesium (Mg), boron (B), etc. Metal particles with different grain sizes considerably influence the combustion and hazardous properties of solid rocket propellants: in particular, the burning rate was significantly enhanced by adding fractions of nano-sized particles, while the rheological performance of propellant slurry was significantly increased as well [3,4]. Several works on nano-sized metal powders were conducted worldwide, and many good achievements were obtained [5,6,7,8]. Replacement of micro-sized particles by nano-sized particles in the propellant composition is one of the most important aspects thanks to the excellent features of energetic nano-ingredients [9]. For example, the effect of Al particle size on the combustion performance of fuel-rich propellants with high Al content was studied. It was found that the presence of aluminum nanopowder (nAl) can evidently improve the combustion performance of propellants [10]. Compared with mAl, nAl burns directly by single particles instead of melting followed by burning, which would decrease the diffusion length and shorten ignition time [11]. Meanwhile, the high surface energy of nAl particles may lead to the agglomeration of Al, which not only inhibits the mass transfer and heat diffusion during the reaction, but also causes poor mechanical properties of the aluminized explosives [12,13]. The qualities of nAl (morphology, size distribution, and presence of alumina (Al_2_O_3_)) have an important effect on energy release [14]. To address these issues, several series of nAl-based coated energetic materials, including Al@polyvinylidene fluoride (PVDF), Al@nitrocellulose (NC), and Al@ammonium perchlorate (AP)/NC, were fabricated via spray coating [11,15], and a novel structure composed of energetic metal-organic frameworks (EMOFs) and polydopamine (PDA)-coated Al (nAl@PDA) was designed to develop energy-storage. The nAl surrounded by metal oxides was activated with self-sustained combustion with multi-level energy-release [16]. Recently, core–shell configuration was introduced to tune the microstructure of energetic composites, which has been demonstrated to be a highly effective strategy to obtain synergistic properties [17,18,19]. In a previous work, energetic polymer GAP was covalently grafted onto the nano-Al surface. The core–shell structured Al@GAP achieved water resistance, enhanced compatibility with polymeric binder, and enhanced combustion performance [20]. In this review, nAl coated with various materials are summarized, and the evaluation of active Al content (AAC) is carried out. The focus of this paper is on the combustion enhancement of different types of nano-sized metal particles, such as nAl, zirconium (nZr), titanium (nTi), nickel (nNi), etc., on solid propellants. The resulting properties are compared with those of propellants loaded with micro-sized Al (mAl) powder. The prospects and future trends of metal nanopowders were investigated, which could make the new propellants useable for solid rocket motor applications.

## 2. Nano Al

nAl, as a new type of metal fuel, is often used as additive in solid propellants due to its unique properties, such as high energy density and low temperature oxidation performance, which can evidently improve the burning rate and possibly decrease the pressure exponent (*n*). For example, see [21,22]. Despite their advantages, such as large surface, small particle size, and high surface energy, nAl powders are easy to react with oxygen to form a dense oxide film (Al_2_O_3_) when exposed to air. The Al_2_O_3_ formation will limit the application of nAl powders in rocket propellants and bring a certain degree of difficulty in storage [23]. Therefore, in order to maintain its activity and storage performance, it is necessary to improve the surface coating of nAl.

### 2.1. Coating of nAl

**Figure 1:**
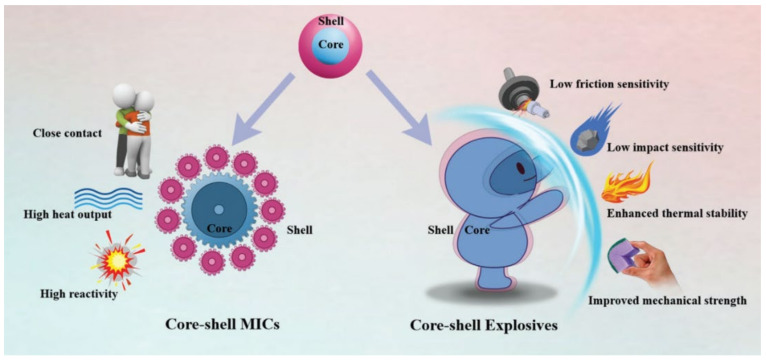
Schematic of core–shell structured nEMs advantages. Reproduced from [24], with permission from Wiley, 2020.

The materials for surface coating of nAl powders are mostly inorganic materials, organic materials, energetic materials (such as carbon, metal, and metal oxide coating), polymer coating, etc. Coating of nAl can form core–shell structures, and core–shell structured nano metals have gained considerable attention due to their improved material properties and combined multiple functionalities. Core–shell structured nano energetic materials mainly include core–shell structured explosives and metastable intermolecular complex (MICs). For core–shell explosives, the “shell” acts as a protective “coat” on sensitive explosives, making them more resistant to external stimuli such as high temperature, friction, impact, static electricity, and compression. For core–shell structured MICs, the fuel and oxidizer are like a well-matched “gear”; they have large and close contact with each other, so that the thermite reaction is easier and more complete than other structured MICs, resulting in increased heat output and improved reactivity. Figure 1 schematically illustrates the advantages of core–shell structured particles.

#### 2.1.1. Carbon Coating

Carbon-coated nAl powder (nAl@C) was first used by Rouff [25]. Generally, carbon is stable at low temperatures, and it can be oxidized to CO_2_ at high temperatures without increasing the load of solid propellant. Thus, it is a good coating agent for nAl powders. For instance, one thin carbon coating using either a laser induced plasma or a DC plasma-arc was investigated, and the carbon coating was created by injecting ethylene (C_2_H_4_) directly downstream of the plasma. The elemental composition of nAl@C was measured in real time with a recently developed quantitative single particle mass spectrometer (SPMS). It was found that the nAl@C features a layer of 1–3 nm thickness [26]. Another example is [27], where nAl@C was prepared under a methane and Ar gas atmosphere using laser-induction complex heating method: a core–shell structure of nanoparticles with 20–40 nm diameters and 3–8 nm thickness was obtained.

#### 2.1.2. Metal Coating

nAl can be coated with metals, such as B, nickel (Ni), etc. When B is used as the surface coating of nAl, the gasification of metal particles can be promoted during the heating process, and an extra exothermic enthalpy of (2.1–4.1) J g^−1^ can also be obtained [28]. For example, when nAl powder is coated with B (nAl@B), the dispersion of nAl@B improves, the onset oxygen temperature increases by 30–40 °C, and the heat of combustion of Al also increases due to the AlB_2_ coating layer. Moreover, the active Al content of nAl@B shows little change after storage in air with 70% humidity for one year [29].

When nAl is coated with Ni, not only can the physicochemical properties of single Al particles be maintained, but the stability and dispersion of nAl powder can also be improved [30]. Moreover, it was found that Nican promote the oxidation of Al. For instance, a core–shell structured nAl@Ni particle was prepared using a displacement reaction method in N_2_ atmosphere for 12 h and nAl powder with 60–100 nm diameter as raw material. It was found that the stability of the resulting nAl@Ni particle was good. The active Al content changes little after storage for one month in air. Moreover, it can increase the burning rate of solid propellants when fractions of Al are replaced by nAl@Ni [31,32]. Furthermore, the Ni layer plays a crucial role in enhancing the oxidation of nA1 powders due to its oxygen transfer ability. For instance, nAl@Ni was prepared by a replacement reduction method, and a larger content of Ni powders further increases the weight growth rate of A1 powders. The ignition and combustion behaviors of composite powders were observed at 1000 °C, when the covering content of Ni increased to 8.93% [33].

#### 2.1.3. Metal Oxide Coating

The composite of nAl and metal oxide forms a kind of high reactive energetic material, which can reduce the agglomeration of particles and increase the burning rate of solid propellants as well. Al_2_O_3_ is one of this kind of metal oxides, because the coating process is simple, and only a small amount of air is needed to be filled to the stored nAl powder in an inert atmosphere. For instance, nAl powder was coated with Al_2_O_3_ (nAl@Al_2_O_3_), and particles with 2–5 nm coating thickness were obtained [34]. However, there are drawbacks, such as the Al_2_O_3_ layer can hinder the heat release behavior of nAl powder in the combustion process and even increase its oxidation exothermic temperature. Moreover, the active Al content could be reduced to 30–50% when the average diameter of nAl powder reaches 20–30 nm. Other coated particles, such as nAl@CuO, nAl@Fe_2_O_3_, nAl@Co_3_O_4_, etc., with high reactivity and quick reactive speed were prepared as well [35,36,37,38].

#### 2.1.4. Organic Acid Coating

As a kind of surface modifier, the carboxyl group of organic acid can not only react with Al to form a chemical bond, which can be firmly coated on the surface of nAl powder, but the carbon chain at the other end can also form a steric hindrance, improving the dispersion and protecting the activity of nAl powders. Moreover, organic acids can be used as a combustion agent to provide energy in the combustion of nAl powders [39]. For instance, oleic acid (OA, C_18_H_36_O_2_) was dissolved in ethanol to coat nAl (nAl@OA) with active Al content of 61.5% for 30 nm diameter particles, and the exothermic peak temperature moved forward to about 500 °C, while the thermal decomposition temperature of nAl@OA/hexogen (RDX) is lower than that of Al/OA/RDX mixture [40,41,42]. The effect of nAl@SA, formed with stearic acid (SA)-coated nAl, on the combustion characteristics of nAl were also studied. Results show that nAl particles disperse more evenly, the mass fraction of Al increases by 9.62%, and the combustion of nAl@SA powder is more complete than that of nAl powder. In particular, the optimized mass proportion of SA and Al is 1:3 [43]. Meanwhile, nAl@OA and nAl@SA were suspended in kerosene and ethanol: it was found that nAl particles with a protecting surface reveal increased stability towards oxidation in air and in water during the storage period, showing for organic-coated metal particles the presence of two layers (an external organic layer and an internal oxide layer) [44,45].

When perfluorooctanoic acid (PA, C_17_H_35_COOH) was introduced to coat nAl by means of the electrical explosion of wires (EEW) method, it was found that the active Al content decreased from 79% to 59% after nAl@PA was stored for one year [46]. Furthermore, nAl was coated with paraffin and PA by liquid phase chemical method, producing spherical particles with coated Al diameter of 80–120 nm and active Al content of 80.8% [21]. Additionally, nAl powder was coated with perfluorotetradecanoic acid (FS) to form nAl@FS under nitrogen atmosphere: it was found that the dispersity of nAl@FS was improved, and the particle size distribution was more homogeneous. Compared with nAl powders, the ignition delay time of nAl@FS is shorter under the same laser heat flux density. The combustion reaction of nAl@FS is more intense while the flame brightness is higher in the laser ignition process. During the combustion of nAl@FS at low pressures, the flame is more intense, and the flame brightness is higher [47]. In another example, the electric spark ignition for a cloud of nAl powder formed in a combustion tube was used to measure comparative flame front propagation velocities with different Al particle sizes with and without organic coating. The results show that nAl particle clouds burn faster than mAl particle clouds. Reducing the size of particles from micro-scale to nano-scale reduces the ignition and burning times significantly and increases the particle cloud flame propagation velocity [48].

#### 2.1.5. Polymer Coating

A binder used to coat nAl powder can isolate the contact between nAl and air. In recent years, NC and azide binder with high energy have become very popular energetic materials. To prevent further oxidation and inactivation in the air, nAl powders were pretreated with silane coupling agents and then coated with glycidyl azide polymer (GAP) under nitrogen atmosphere, and thus GAP-coated nAl (nAl@GAP) was obtained [49]. The results show that the coupling agent plays a role as a bridge between GAP and nAl: the core–shell particles are observed, the stability in hot water and energy releasing performance of nAl improve with the assistance of GAP, and the thickness of the GAP shell layer could be tuned by changing the relative ratio of reactants [20,50]. While nAl and nAl@GAP have little influence on the liquefaction temperature of ADN, the decomposition temperature increases significantly. Several nAl coatings with polymer make the activation energy of nAl increase slightly, while it has little influence on the combustion of propellant strands and solid motor charges. For instance, hydroxyl terminated polybutadiene (HTPB) was used to coat nAl to form nAl@HTPB. When HTPB coating ratio was 21.6%, the exothermic heat of Al in the sample is 4.954 kJ·g^−1^, the active Al content is less than 50%, and the activation energy is 253.21 kJ·mol^−1^ [51].

**Figure 2:**
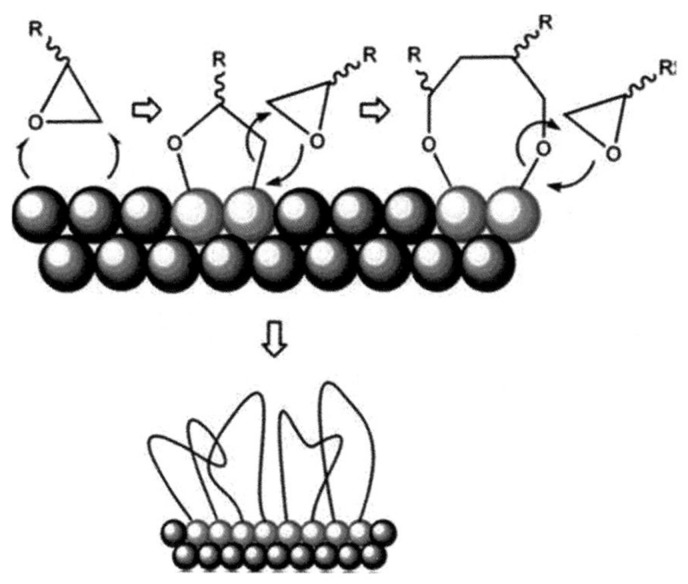
Schematic diagram of nAl powder coated with epoxy compound. Reproduced from [23], with permission from Applied Surface Science, 2007.

Being NC a very important energetic binder in explosives, nAl powders were coated with it (nAl@NC), and the performance was compared with that of nAl@OA (oleic acid, C_17_H_33_COOH), nAl@SA (stearic acid, C_17_H_35_COOH), and non-coated particles. It was found that the surface protection of nAl powders by the above chemical origin coatings leads to some advantages in practical applications of energetic systems (Figure 2). In fact, nAl powders with a protected surface show an increased stability to oxidation in air during the storage period and higher reactivity under heating [52]. By using a mechanical chemistry method, it was also found for Al coated with NC and TNT that nAl@NC+TNT has high thermal reaction characteristics. When coated particles were added to the RDX/paraffin/Al with 76/4/20 mass ratio, the activation energy, impact sensitivity, and heat of explosion increased by 8.5%, 9.1%, and 6.48%, respectively, while the friction sensitivity decreased by 2.4%.

#### 2.1.6. Energetic Materials Coating

RDX, which has the advantages of high specific impulse and low combustion temperature, is the main nitramine filler in solid propellants. RDX-coated nAl powder (nAl@RDX) can improve the performance of Al powder in solid propellants and reduce the activation energy. For instance, nAl coated with RDX particles were prepared using a solvent–antisolvent method with N,N-methylformamide (DMF) and toluene as the solvents. It was found that the active Al content of nAl@RDX (89%) is higher than that of Al/RDX mixture (66%). Moreover, the initial decomposition temperature of RDX decreased by 45 K by the catalysis of nAl [53].

#### 2.1.7. Fluoride Coating

**Figure 3:**
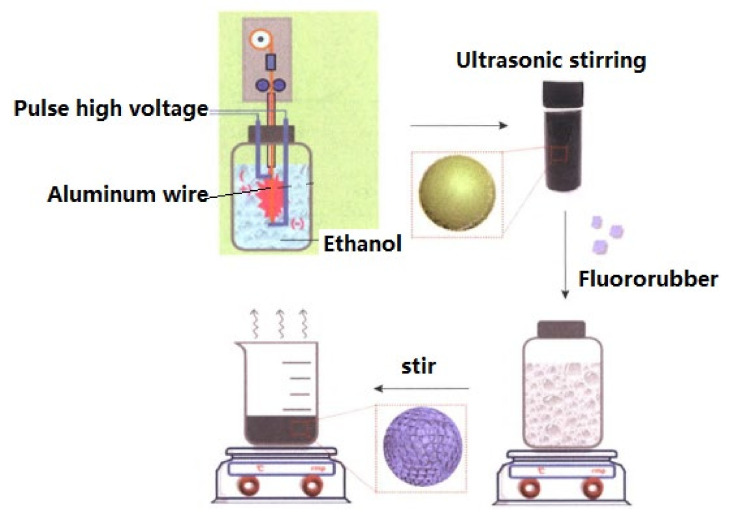
Preparation process of coated nAl powders. Reproduced from [56] with permission from Acta Armamentarii, 2019.

Based on theoretical calculations, fluorine was proposed as an additional oxidizer to change the reaction rates and Al combustion mechanisms. A typical preparation process is shown in Figure 3 [54]. It was found that the underwater explosion energy increases with increasing fluorine content in the binder, while it decreases with increasing binder content [55]. Thus, nAl powders prepared by EEW method were coated with 5%, 10%, and 15% of fluororubber (nAl@F). Results show that the specific surface area of the nAl@F powders is slightly larger than that of the passivated nAl powders, while the active Al content is up to 85.85%. Fluororubber coatings chemically adsorb on the surface of nAl powder, which enhances the thermal stability of nAl and prevents further oxidation. The fluororubber also promotes the combustion of nAl powders, and the heat of combustion of the nAl@F is higher than that of nAl powders.

The effect of nAl@F on the combustion properties of HTPB-based fuels was also investigated [57]. The results showed that nAl@F powder has a certain promoting effect on the regression rate of the fuel, and this effect is independent of the oxidizer mass flux. The regression rate of the fuel with nAl@F is 13% higher than that of the fuel without nAl@F over the oxygen mass flux range of 100–380 kg·m^−2^·s^−1^. In another instance, the effect of morphology, structure, and active Al content of the coated nAl particles with phenolic resin (PF), fluororubber (Viton B), and shellac through a solvent/nonsolvent method were investigated. The results indicated that the coated nAl particles have core–shell structures, and the thickness of the coating film is 5–15 nm. Viton B coating has a much better protective effect by the active Al content. The energy amount and energy release rate of nAl@PF, nAl@Viton B, and nAl@shellac particles were larger than those of the raw nAl [58].

#### 2.1.8. Other Materials Coating

In some cases, propellant ingredients were used as coating materials: under these circumstances, the ignition temperature could be reduced, and the heat release could be enhanced. For example, AP is a strong oxidant, which is commonly used in solid propellants or explosives. For AP-coated nAl (nAl@AP) obtained using recrystallization method, the ignition temperature of nAl@AP was less than that of nAl at a heating rate of 20 °C·min^−1^. In particular, for nAl@AP with 10% and 15% of AP, the ignition temperatures decreased by about 200 °C, which is due to the rapid decomposition of AP, and has an obvious effect on nAl ignition [59,60]. Dioctyl sebacate (DOS) is another common ingredient of propellant: when DOS coating ratio is 29.8%, the exothermic heat of Al in the sample is 4.955 kJ·g^−1^, while the active Al content is 75.6% [61]. When the mass concentration of dopamine (DA) is 3.5 g·L^−1^, DA can be polymerized into a firm PDA coating on the surface of nAl (nAl@PDA), and the crystal form of Al remains unchanged before and after coating [62].

The effect of differently coated nAl (including nAl@NDZ with 5% of NDZ, nAl@NGTC with 5% of NGTC, nAl@ polymer with 50% of polymer, and nAl@Al_2_O_3_ with 1% of Al_2_O_3_) on the combustion performance of composite propellant (AP/Al/HTPB = 70/15/15, with 5% of mAl replaced by nAl) were investigated [63]. It was found that the active Al content and heat of explosive decreased from 95.33% and 5540.9 J·g^−1^ of mAl to 55.74–67.38% and 4388.7–4784.3 J·g^−1^ of nAl, respectively. However, the burning rate at 7 MPa increased from 9.31 mm·s^−1^ to 10.79 mm·s^−1^, while the pressure exponent decreased from 0.48 to 0.22. It can be concluded that the addition of nAl is beneficial to improve the burning rate and possibly reduce the burning rate pressure exponent, but at the same time, the low active Al content of nAl powder leads to a reduction in the propellant explosive heat. This is in broad agreement with [22].

### 2.2. Evaluation of Active Aluminum Content (AAC)

**Figure 4:**
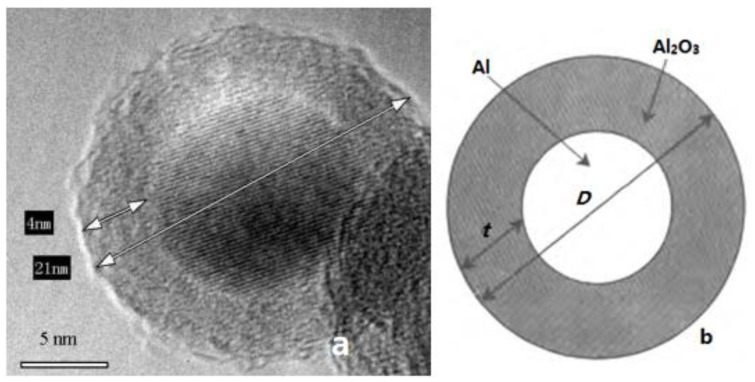
Images of nAl: (**a**) high resolution TEM image of nAl; (**b**) structure diagram of nAl powder. Reproduced from [71] with permission from Huazhong University of Science and Technology, 2008.

Adding an appropriate amount of nAl powder can significantly improve the performance of solid propellants. However, due to the small size and surface effect of nAl powder, its reactivity is very high. Once nAl is prepared, the surface atoms will be oxidized, resulting in a reduction in Al content. Therefore, research on nAl powder reactivity, that is, how to maintain or control its reactivity, is of great significance to researchers worldwide. At present, the commonly used determination methods of elemental Al content mainly include gas volumetric method [64,65], redox titration method [66], thermogravimetric analysis method [67], and transmission electron microscopy method [68]. Taking transmission electron microscopy (TEM) as an example, this method first assumes that the structure of nAl powder is spherical and then uses high-resolution transmission electron microscope photos to evaluate the average particle size and oxide layer thickness of nAl particles through proportion calculation, as shown in Figure 4. The Al content of nAl powder is obtained from Equation (1) [69,70] as

(1) $$C_{Al}=\frac{\rho_{Al}\cdot {\left(D-2t\right)}^{3}}{\rho_{Al}\cdot {\left(D-2t\right)}^{3}+\rho_{Al2O3}\cdot \left[D^{3}-{\left(D-2t\right)}^{3}\right]}\times 100\%,$$

where, *D* is the average size of nAl particles including the oxide layer; *t* is the oxide layer thickness; and $\rho_{Al}$ and $\rho_{Al2O3}$ are the density of Al and Al_2_O_3_, respectively.

**Figure 5:**
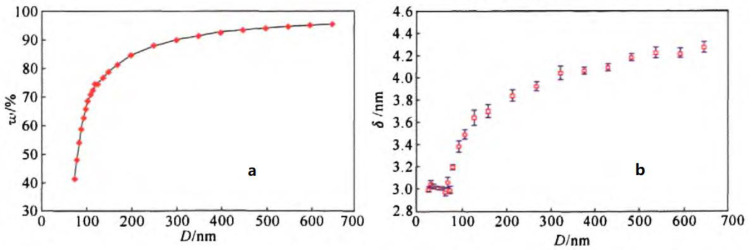
Curve of active Al content and oxide film thickness vs. diameter of nAl powder. (**a**): Active Al content vs. nAl diameter; (**b**): Oxide film thickness vs. nAl diameter. Reproduced from [72] with permission from Chinese Journal of Explosives and Propellants (Huo Zha Yao Xue Bao), 2011.

In addition, the relationships between particle size and active Al content (Figure 5a) as well as particle size and oxide layer thickness (Figure 5b) were obtained. When the particle size is less than 70 nm, the oxide thickness remains basically unchanged, and the particle size is less affecting the oxide thickness of Al powder. When the particle size of Al powder exceeds 70 nm, the oxide layer thickness increases rapidly. At this time, the increase in particle size plays a decisive role in the oxide layer thickness of Al powder. This finding indicates that there is a lower limit on the thickness of nAl oxide film [72].

**Figure 6:**
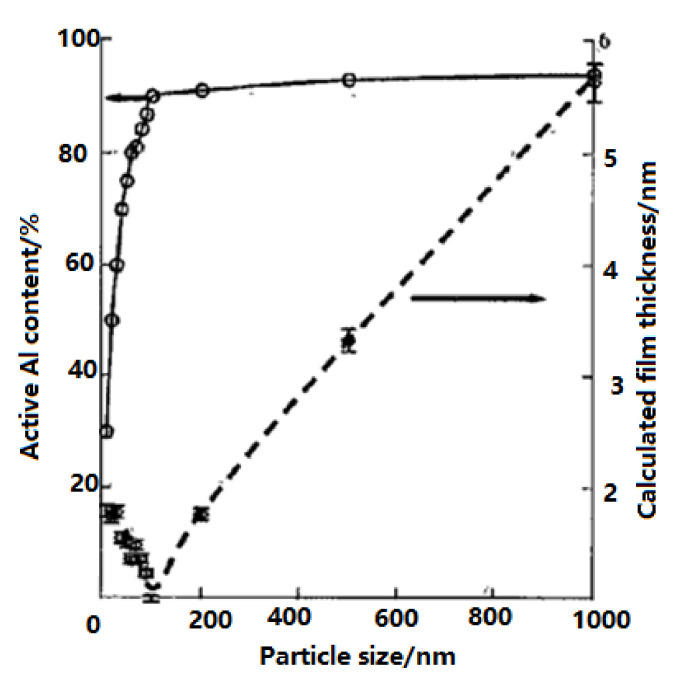
Relationships between active Al content and calculated oxide layer thickness vs. particle size. Reproduced from [74] with permission from Russian Journal of Physical Chemistry B, 2010.

Furthermore, with decreasing nAl particle size, the oxidation initial temperature decreases, but the oxidation heat release shows a downward trend [32,73]. For example, the relationship between active Al content, particle size, and oxide layer was studied in [74]; see Figure 6. It was found that, when the nAl particle size is less than 100 nm, the activity decreases sharply and the proportion of oxide layer increases. When the nAl particle size is 10 nm, the AAC is only 30% and grows to about 30–50% when the particle diameter increases to 20 nm–30 nm. When the particle size of Al powder is greater than 200 nm, the AAC remains at 90%. With the increase in particle size, the thickness of oxide layer continues to increase, but the activity is not significantly improved. It can be seen that, for particle diameter around 100 nm, the nAl powder is relatively stable, and the Al content is high.

**Figure 7:**
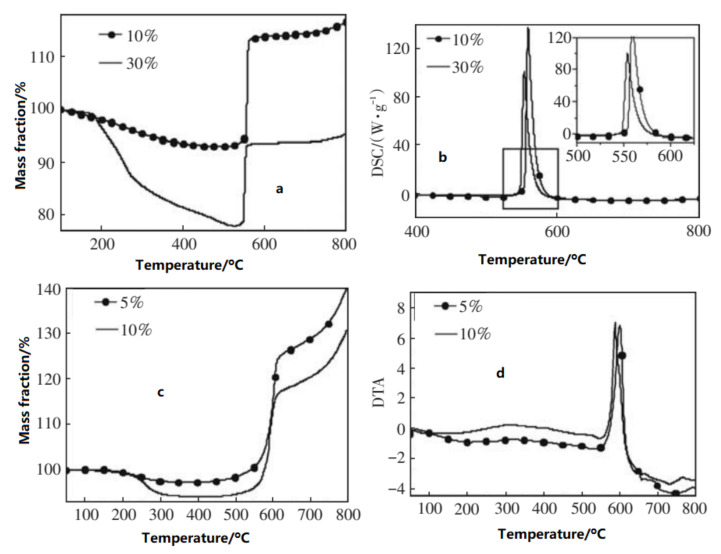
TG–DTA curves of nAl powder with different mass fraction of coating. (**a**): 10% and 30% coating with 50 nm; (**b**): 5% and 10% coating with 100 nm; (**c**): Mass fraction of TG curves; (**d**): DTA curves. Reproduced from [76], with permission from Journal of Solid Rocket Technology (Gu Ti Huo Jian Ji Shu), 2011.

Based on the coating of nAl with different materials and contents, the active Al content would be influenced by the coating layer. For example, the thermal properties of several kinds of nAl coated with polymer of different shell and thickness were studied by TG-DSC. The shell’s effect on the reactivity of nAl was analyzed [75]. The results show that, for nAl with the same particle size, the thicker the shell is, the more weight the particle loses before oxidation and the less weight the particle gains during oxidation. From 520 °C to 800 °C, 50 nm Al powder with 10% and 30% polymer coating gains 23.5% and 17.4%, respectively. Additionally, Al powder with 100 nm diameter coated by 5% and 10% polymer increases 42.5% and 36.5%, respectively. The energy release for 50 nm Al powder is quicker than that of 100 nm Al. Al powder of 50 nm reaches the max oxidation rate at 550 °C, much lower than that of 100 nm Al powder (590 °C), but the reactivity of 50 nm Al powder is lower than that of 100 nm Al powder (Figure 7).

Additionally, the long-term storage stability and self-activation reaction capability of nAl coated with 1H, 1H, 2H, 2H-perfluorodecyltriethoxysilane (FAS-17) were studied. In terms of energetic performance, compared to the two-step slow oxidation of nAl, the heat-release rate of nAl@FAS-17 is significantly enhanced, resulting in a drastic oxidation process profiting from the surface reaction between the FAS-17 and Al_2_O_3_ layer. More importantly, the ignition and combustion properties of nAl@FAS-17 are also greatly improved, which can undergo self-propagation combustion with a fairly high energy output even after storage in water [77].

### 2.3. Combustion Enhancement of nAl on Solid Propellants

The burning rate of propellant determines the rate of gas generation, which determines the pressure inside the motor and the overall thrust [21]. Various factors such as the particle diameter, oxidizing species, pressure, and temperature affect the burning rate of the particles [78,79]. It was reported [80] that the initial oxidation temperature of nAl is much lower than that of mAl powder and the mass increment for nAl is higher than that of mAl at each oxidation stage, which may be attributed to the smaller particle size and larger specific surface area of nAl compared to that of mAl powder. The burning rate of nAl powder itself is higher than that of mAl powder, the ignition threshold of nAl is lower than that of mAl powder, and the difference increases with decreasing nAl particle size [22].

Moreover, the ignition temperature of nAl is lower and reactivity is higher than that of micro-sized Al. On one hand, the lower ignition temperature can make nAl powder oxidized and release heat over the low temperature range of 500–600 °C. On the other hand, the higher reactivity of nAl can shorten the ignition delay and combustion time, and the particle size of condensed combustion products (CCPs) is small, and there is much heat feedback to the propellant burning surface [81].

#### 2.3.1. nAl Effects on the Combustion Performance of HTPB-Based Composite Propellants

The effects of coated nAl (20–50 nm) on the burning rate and pressure exponent of HTPB composite propellants (HTPB/AP/Al = 14.5/65.5/20) have been investigated; 3%, 6%, and 9% mass fraction of mAl were replaced by coated nAl and compared with that of mAl powder. The results show that the burning rates of propellants increase as nAl content increases, but the pressure exponents over the pressure range of 3–7 MPa decrease. Compared with propellant containing mAl, the increments of burning rates of propellants containing nAl powder reduce gradually with increasing pressure due to the combustion characteristics and ignition performance differences of nAl and mAl powder [82]. Meantime, the combustion features of 85% bimodal HTPB/AP/Al composite propellant with 0.75% by mass of nAl into wet-mixed boron at varying ratios of Al to boron (4:1, 2:3, 1:4) were performed between 3.45 and 15.51 MPa in a constant volume pressure vessel using nichrome wire ignition and compared to that of formulations with dry magnesium-boron (MgB) and mAl@B at the same mass loadings. Results showed the dry-powder nAl with wet-mixed boron and MgB formulations produced modest increases in burning rates compared to the composition without metals. The most promising formulation in terms of burning rate improvement was the 0.15% boron and 0.60% Al-coated particles, which increased the burning rates by 54% [83].

Furthermore, the nAl effects on the performance of fuel-rich propellant (HTPB/Al/Mg/AP/additives in the mass ratio 22/20/21/35/2) were investigated. The burning rate and pressure exponent of the propellants with and without nAl particles were obtained under different pressures for a series of formulations over the range 0–20% nAl. It was found [22] that the burning rates of fuel-rich solid propellants increase with increasing pressure, and the increase in extent over the pressure range 0.5–1 MPa is higher than that over the pressure range 2–3 MPa. The burning rate increases by 77% for fuel-rich propellant loaded with 20% of nAl powder at 1 MPa. The propellant pressure exponent increases a little for increasing mass fraction of nAl powder over the explored pressure range. By comparison, the pressure exponent of the formulation without nAl is 0.38 (0.5–3 MPa), which is the lowest one in the tested series.

Additionally, the densities and heat of combustion of fuel-rich propellants were measured and compared with the theoretical data. The density of fuel-rich solid propellants with different nAl mass fraction is in the range 1.627–1.636 g·cm^−3^, which is lower than that of the propellant with mAl powder (1.641 g·cm^−3^). The measured heat of combustion of fuel-rich propellant increases a little when a given mass fraction of mAl is replaced by nAl in the formulation, indicating that the combustion efficiency of nAl powder is higher than that of the mAl to some extent. At the same time, it was also found that the friction sensitivity and impact sensitivity of fuel-rich propellant increase with increasing mass fraction of nAl powder: the increasing slope of samples with nAl powder over the range 0–5% mass fraction is much higher than that of the remaining 5–20% interval, while the impact sensitivity of samples decreases significantly when the nAl mass fraction falls in the same interval 5–20% [84].

#### 2.3.2. nAl Effects on the Combustion Performance of NEPE Propellants

**Figure 8:**
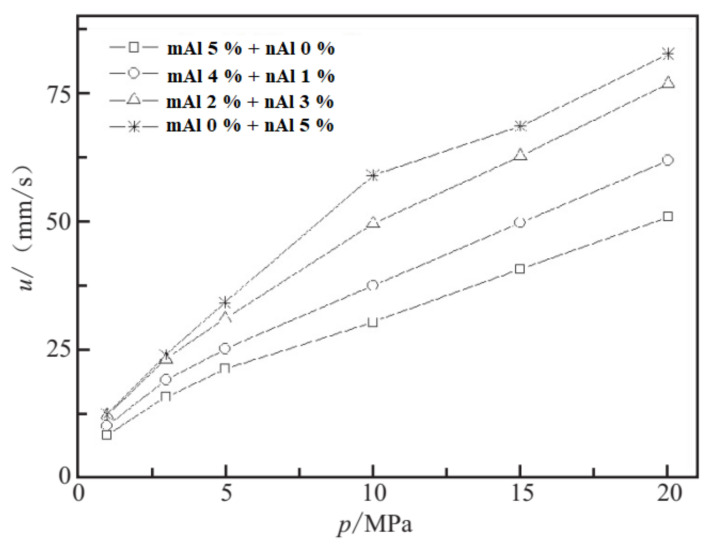
Effect of nAl and mAl on the combustion performance of NEPE propellants. Reproduced from [85] with permission from Journal of Solid Rocket Technology (Gu Ti Huo Jian Ji Shu), 2014.

The effect of nAl particles on the combustion performance and heat of explosion of NEPE solid propellants (PET/NG+TEGDN/AP/HMX/Al = 7/18/35/35/5 in mass fraction) is presented in Figure 8. Compared to mAl particles, nAl particles show the advantages such as high reactivity, low ignition energy, and strong heat feedback to the propellant combustion surface, which increase the burning rate significantly by 1.5–2 times (1–20 MPa), while the pressure exponent first increases from 0.57 to 0.68 (1–10 MPa) and then decreases 0.75 to 0.49 (10–20 MPa). It was also observed that the flame appears slightly dark for NEPE propellant with 5% mass fraction of mAl particles at 1 MPa, with a few bright lights during the combustion process, which are attributed to the presence of Al powder. The flame height increases with increasing nAl mass fraction, and it is the highest for 5% nAl. When the pressure increases to 3 MPa, the combustion is more vigorous, the dark area disappears, and the flame spits out from the burning surface. Meantime, the heat of explosion of NEPE solid propellants does not show much change with increasing nAl mass fraction in the formulation. The heat of explosion of the sample with 5% mAl replaced by nAl is similar to that without nAl.

#### 2.3.3. nAl Effects on the Combustion Performance of CMDB Propellants

**Figure 9:**
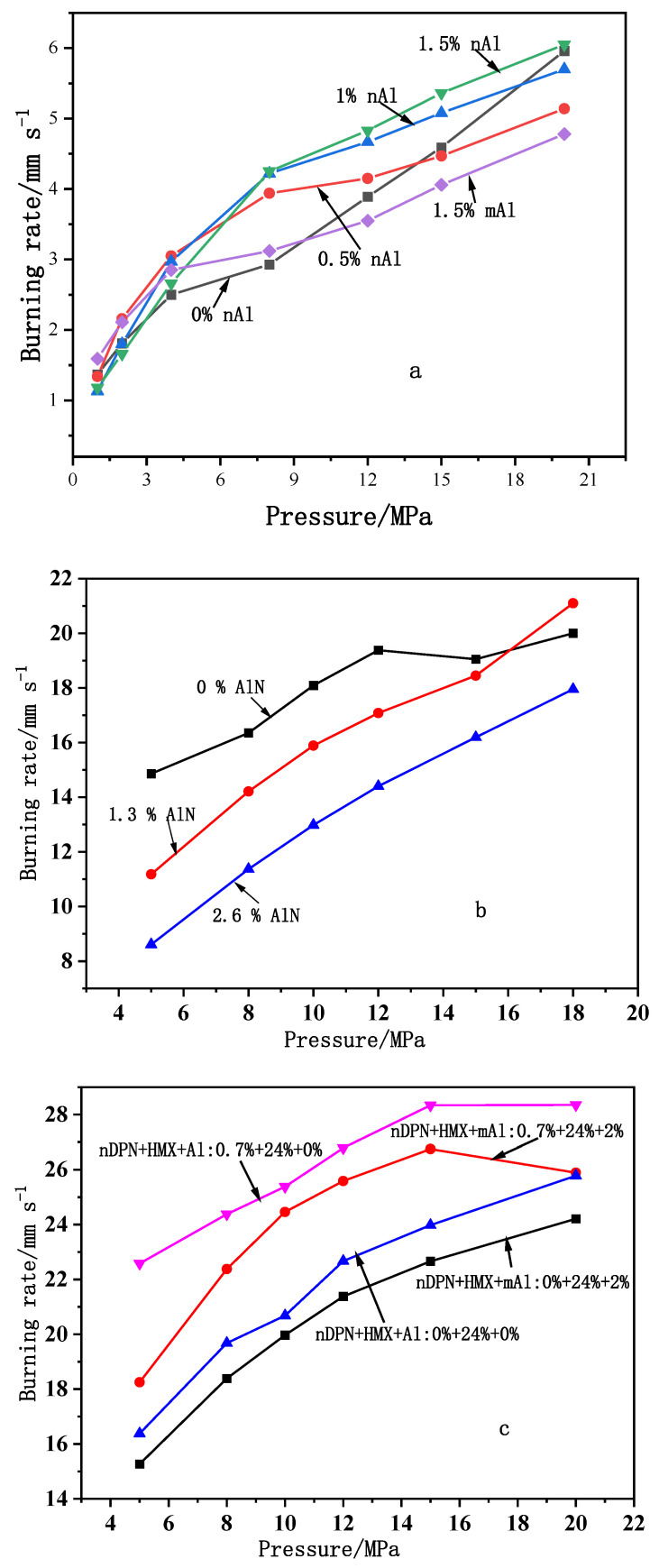
The burning rate vs. pressure curves of RDX-CMDB propellants with different nano powders and contents. (**a**)—73.5% NG/NC + 19.5% burning rate inhibitor + 4.0% catalyst + 3.0% additives with and without nAl; (**b**)—63.0% NG/NC + 2.3% catalyst + 2.8% additives + 26% RDX + 4.6% diethyl phthalate (DEP) + 2.6% (nAl+Al_2_O_3_) with and without nAlN; (**c**)—63.4% NG/NC + 5.85% + 4.75% additives + 24% HMX with and without nDPN. Reproduced from [86], with permission from Chinese Journal of Explosives and Propellants (Huo Zha Yao Xue Bao), 2013.

The effects of nano-sized composite, such as nano composites DPN (nDPN), nano-AlN (nAlN), and nAl on the combustion properties of DB propellant, CMDB propellant with RDX, and CMDB propellant with HMX/Al, are shown in Figure 9. It can be found that the burning rate of formulation with 0.5% mass fraction of nAl at 2–12 MPa is higher than that of formulation without nAl. With the increase in the content of nAl, the burning rate of propellant increases, which may be because the heat feedback from the flame increases with an increase in the nAl content. Meanwhile, the pressure exponent is low over the pressure range of 8–20 MPa. Compared with the same content (1.5%) of mAl, the burning rate of the propellant increases significantly, which may be due to the higher heat feedback of nAl with respect to mAl powder (Figure 9a).

When nAlN was used to replace Al_2_O_3_, the propellant burning rate at 5–15 MPa decreased by an average of 2 mm·s^−1^. The burning rate of the formulation with 1.3% of nAlN at 18 MPa was higher than that without nAlN, and the pressure exponent over the pressure range of 5–18 MPa was higher than that of nAlN. The burning rate of propellant with 2.6% of nAlN decreased by an average of 5 mm·s^−1^, while the pressure exponent increased (Figure 9b). Therefore, nAlN looks a good burning rate reducer, which can decrease the burning rate of propellants greatly, and the burning rate decreases more with a rise in content, but it will increase the pressure exponent. The reason may be that AlN is an inert material, which does not participate in the combustion, while it absorbs the heat generated by the propellant combustion. Moreover, addition of nDPN to the formulation can increase the burning rate and pressure exponent over the low-pressure range, especially when the mass fraction of nDPN is less than 1%; notice that the burning rate could be increased greatly (Figure 9c).

Considering all the combustion experiments, nAl powder, for its many advantages, has significant positive effects on the combustion of solid propellants. Compared to mAl powder, a large number of spaces exist between the micro-sized particles for its large specific surface area during the process of particles, such as friction or impact. The air absorbed in the space comes into “hot spot” when compressed, and the increasing temperature of the “hot spot” urges the decomposition of condensed AP and the condensed reaction between AP with nAl powder, until finally, deflagration occurs [87]. From the viewpoint of heat transfer, the addition of nAl powder in the propellant can effectively increase the heat adsorption in the combustion process. From the viewpoint of dynamics, nAl powders can contact polymer binder and gaseous reactants because of their large specific surface area. Additionally, the heat release and heat transmission at the combustion surface for nAl are higher than the values for mAl at high pressure range [87,88,89].

## 3. Nano Ni (nNi)

**Figure 10:**
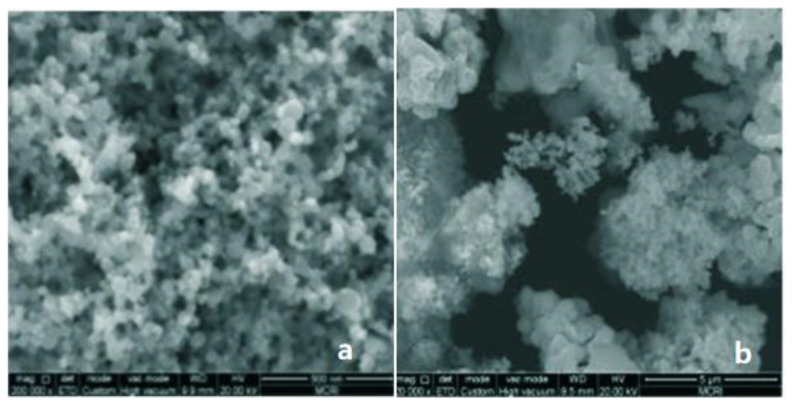
SEM images of several particles. (**a**) nNi; (**b**) mNi. Reproduced from [90] with permission from Chinese Journal of Explosives and Propellants (Huo Zha Yao Xue Bao), 2016.

Nickel nanopowder (nNi) has a series of special physical and chemical properties. It is widely used as catalytic material, battery material, cemented carbide material, and magnetic material. It was demonstrated that nNi has significant catalytic effects on the thermal decomposition characteristics of AP. The exothermic high temperature peak of AP loaded with 5.0% mass fraction of nNi is advanced by 105 °C, which is more effective than the addition of micrometric nickel powder (mNi). The scanning electronic microscopy (SEM) images of three nanometric materials are shown in Figure 10. The particles of nNi were even and globe-shape.

**Figure 11:**
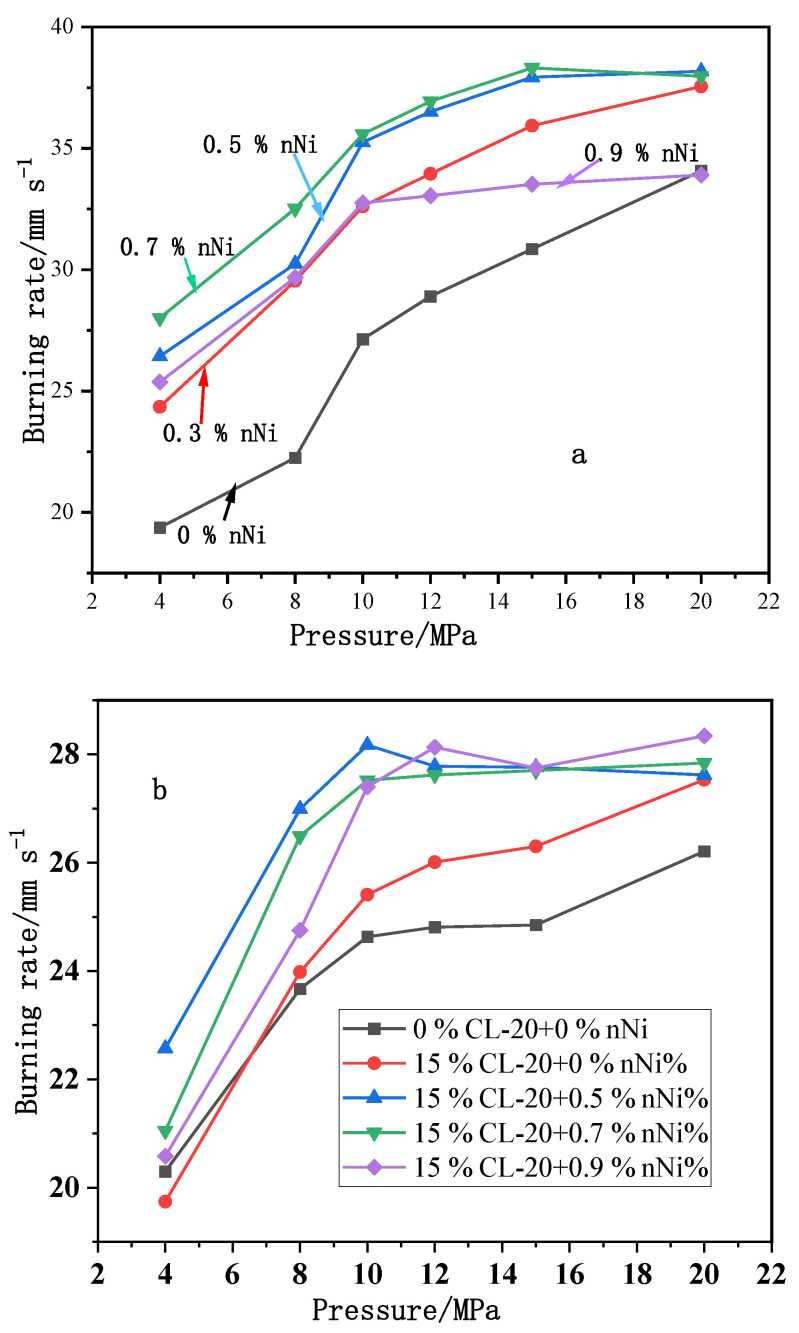

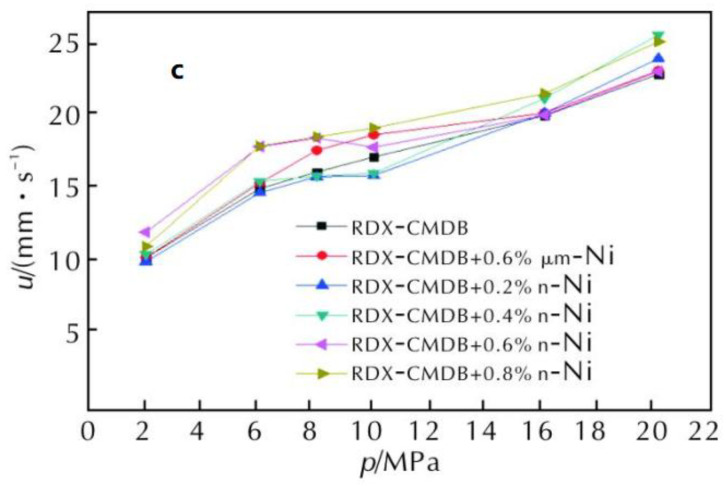
Effect of nNi on the combustion performance of CMDB propellants. (**a**) Formulation with different types of metal nanopowders; (**b**) CL-20-CMDB propellants formulation with different mass fraction of nNi; (**c**) RDX-CMDB propellants with different nNi contents. Reproduced from [90,91], with permission from Chinese Journal of Explosives and Propellants (Huo Zha Yao Xue Bao), 2016 and 2019.

The effects of nNi with 50 nm diameter on the combustion features of CMDB propellants were explored. It was found that addition of nNi to the propellant composition could increase the burning rate and reduce the pressure exponent. When adding 0.7% mass fraction of nNi to Al-CMDB formulation, the propellant burning rate reaches 35.59 mm·s^−1^ at 10 MPa, the pressure exponent reduces from 0.43 to 0.17 over the pressure range 8–20 MPa, and there is a “mesa effect” at 15–20 MPa. When adding 0.5% mass fraction of nNi to CL-20-CMDB formulation, the propellant burning rate increases greatly, and the pressure exponent at 8–20 MPa is 0.1 (Figure 11b). At the same time, the effects of different nNi mass fractions (0.2%, 0.4%, 0.6% and 0.8%) on the combustion properties of RDX-CMDB propellant (NC + NG 61%, RDX 26%, catalyst 4.8%, others 8.2%) were investigated and compared to the baseline formulation. It was found that adding 0.2% nNi can reduce the burning rates of RDX-CMDB propellant at 2–10 MPa; at 10 MPa, the reduction was of 1.26 mm·s^−1^. However, the burning rates at 16 and 20 MPa were increased by 0.18 mm·s^−1^ and 1.07 mm·s^−1^, respectively. With increasing the content of nNi from 0.2% to 0.8%, the propellant burning rate increased at 6–16 MPa, and a maximum increase was achieved at 10 MPa; the propellant burning rate reached the highest value when the mass fraction of nNi was 0.6%. It can be concluded that nNi has a better effect on enhancing the burning rate at low and middle pressure zone of RDX-CMDB propellant than mNi. Additionally, nNi can reduce the pressure exponent between 6–16 MPa more effectively than mNi too (Figure 11c).

In addition, the effect of nNi on the energetic property, thermal stability, mechanical sensitivity, mechanical performance, inner ballistic properties, and the dependence of burning rate on the aging process of CMDB propellant (NC + NG/Ct/Al/nNi + RDX + others = 83/5.75/5.5/5.75 in mass fraction) were studied (Table 1) [92]. Results show that the addition of nNi can slightly improve the mechanical sensitivity of propellant. After adding nNi, the detonation heat of CMDB propellant decreased by 16 J·g^−1^, and the change in specific volume and density was not obvious, while the heat of explosion of propellant with 10% RDX and 0.7% nNi decreased by 9.0 J·g^−1^, and the density decreased by 0.006 g·cm^−3^, but the specific volume increased by 12 L·kg^−1^.

**Figure 12:**
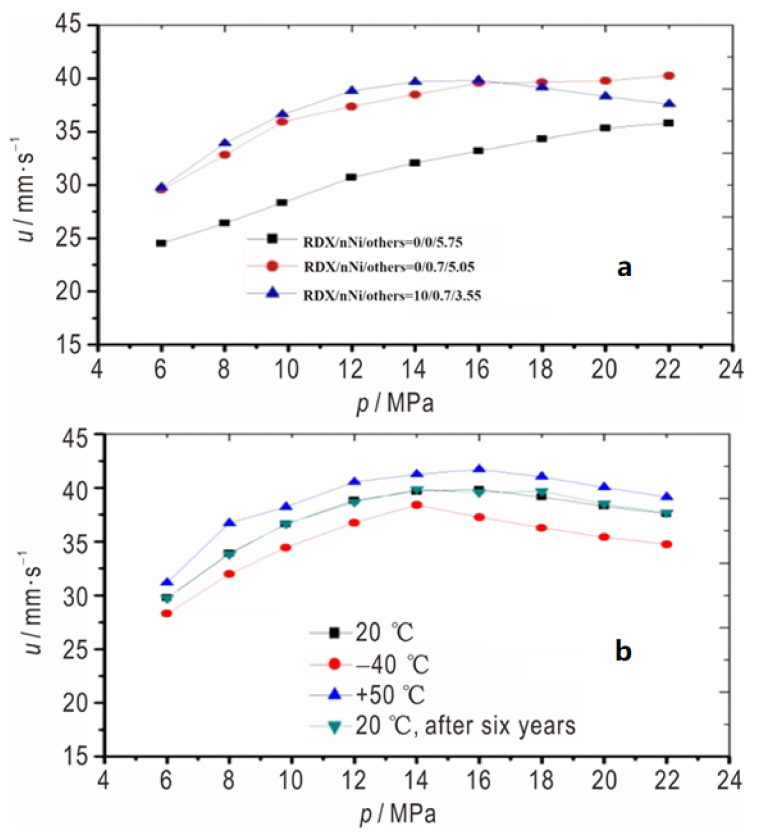
The effect of nNi on the combustion performance of CMDB propellants. (**a**): samples at 20 °C; (**b**): sample RDX/nNi/others = 10/0.7/3.55 at 20 °C, −40 °C, 50 °C, and 20 °C after six years. Reproduced from [92] with permission from Chinese Journal of Energetic Materials (Han Neng Cai Liao), 2019.

The effect of nNi on the combustion performance of CMDB propellants with and without aging at different initial temperatures were tested. It was found that the inclusion of nNi increases the burning rate of Al-CMDB propellant from 28.32 mm·s^−1^ to 36.63 mm·s^−1^ at 9.81 MPa and lowers the pressure exponent over the pressure range of 12–22 MPa from 0.26 to 0.12 (Figure 12).

**Figure 13:**
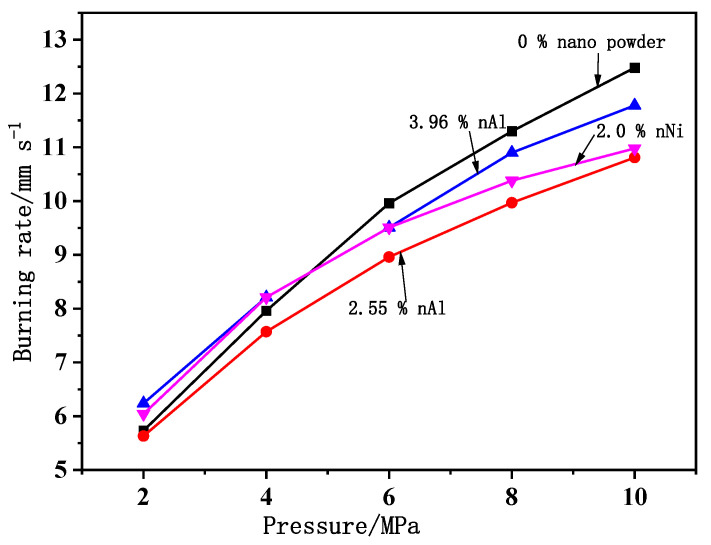
Effect of nano metal powders on the combustion properties of composite propellants. Reproduced from [93] with permission from Journal of Propulsion Technology (Tui Jin Ji Shu), 2004.

Additionally, the effect of nano metal powders on the burning rate of composite propellant with 60/5/15/20 mass fraction of AP/Al/HTPB/RDX was studied. It was found that the addition of 2% nNi or the usage of bimodal Al with a mass ratio of 4:1 nAl:mAl or the addition of 2% nNi powders can improve the combustion of propellant effectively, while the usage of blending of 1:1 nAl:mAl worsens the combustion performance. The ignition threshold and combustion time from the initial particle size to burnout of nAl and nNi are lower than that of mAl. Furthermore, nAl inclined to burn in single mode and nNi has the greatest influence on the thermal decomposition of AP. The results also confirm the high reactivity of nAl, which leads to a lower reaction temperature and higher degree of reaction ratio as compared with mAl in air (Figure 13).

## 4. Nano Ti (nTi), Nano Zr (nZr), and More

The burning rate data of propellants containing different nano-sized particles obtained under different pressures were collected. The effects of different nano metal powders (nAl, *d*_50_ = 51 nm; nTi, *d*_50_ = 83 nm; nZr, *d*_50_ = 64 nm) on the performance of composite propellant (HTPB/AP/Al/Additives = 12/71/15/2 in mass fraction, with 10% of mAl replaced by nano powders) were investigated. It was found that the burning rates of propellant with nAl powder increase with larger pressures and the increasing extent over the pressure range 1.0–7.0 MPa is obviously higher than that over the pressure range 7.0–13 MPa. The pressure exponent of the nAl formulation is 0.33 (1–15 MPa), which is the lowest one among the tested samples. Moreover, comparing the samples with and without nAl powder indicates that the propellant burning rates increase effectively with decreasing size of the metal particles. Furthermore, the density of composite propellants with different nano–sized metal particles is in the range of 1.732–1.784 g·cm^−3^, which is larger than that of the propellant with standard Al powder (1.731 g·cm^−3^). Maybe this is the effect of Al oxide on the surface of nAl. The heat of explosion of composite propellant without nAl is 6045 J·g^−1^, which is higher than that of the samples with nZr and nTi particles (5793 and 5821 J·g^−1^) but lower than that of the sample with nAl particles (6104 J·g^−1^). The mechanical sensitivity result reveals that the use of micro-sized powder leads to a decrease in the sensitivities of friction and impact for solid propellants, while the compositions with nano-sized particles show high friction sensitivity and impact sensitivity [94,95].

**Figure 14:**
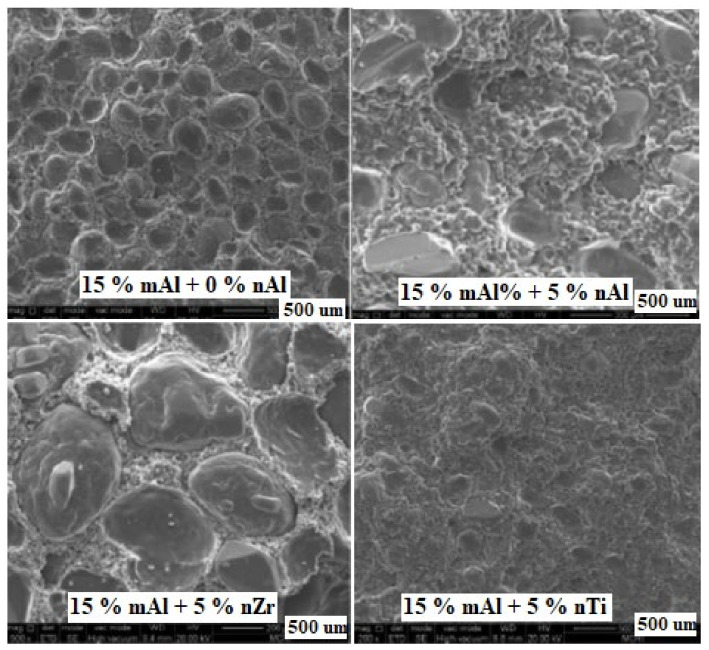
Microstructure surface of composite propellants containing different nano-sized particles. Reproduced from [96], with permission from Wiley, 2014.

From the surface image (Figure 14), lots of granulated particles are visible on the surface of the cured composite propellants. The different nano-sized particles are compatible with the ingredients of composite solid propellant systems, and the granulated particles with smaller diameters can sufficiently fill the spaces between the bigger grains.

For the other nano-sized metal powder, the effect of spherical shaped nano-sized Fe (nFe) with 50 nm diameter, nano-sized Co (nCo) with 42 nm diameter, and nNi with 36 nm diameter powders on the thermal decomposition of AP and combustion properties of HTPB/AP propellant were investigated [97]. It was found that 2% content of nCo powder reduces the high-temperature decomposition peak of AP by 144.0 °C. When the content of nCo increases to 7%, the high-temperature exothermic peak temperature is advanced by 154.7 °C, but the maximum activation energy is increased by 776 J·g^−1^. When the content of nNi is 5%, the high temperature thermal decomposition peak of AP is reduced by 116.7 °C. When the content of nFe is 2%, the high temperature thermal decomposition peak temperature of AP is advanced by 103.2 °C. Furthermore, adding nCo powder, the propellant burning rate increases by 18.35% at 7 MPa and 10.25% at 10 MPa, while the pressure exponent decreases by 17.89% over the pressure range 5–11 MPa. Adding nFe powder, the propellant burning rate increases by 11.52% at 7 MPa and 9.20% at 10 MPa, while the pressure exponent decreases by 14.34% over the pressure range of 5–11 MPa. Adding nNi powder, the propellant burning rate increases by 5.21% at 7 MPa and 1.63% at 10 MPa, while the pressure index decreases by 7.34% over the pressure range of 5–11 MPa.

In summary, to promote the practical applications of nano metals in solid propellants, much investigation needs to be done in the future, such as spheric coating, high-active metal-based composites, active metals, etc. Understanding of these effects opens the path to improved ballistic performance, which will be further investigated. Table 2 summarizes the advantages and disadvantages of the examined nano metals on the performance of energetic compositions.

## 5. Prospects and Future Trends of Metal Nanopowders

For Al nanopowders, the steady burning rates are not affected by particles in the micrometric range (Brunauer–Emmett–Teller (BET) ≤ 2 m^2^ g^−1^) but strongly increased by decreasing size particles over the explored nanometric range; for very small particles, the appreciable decrease in active metal hinders a further increase in steady burning rate. Pressure exponents showed only minor changes within the limits of strand burner experiments conducted under standard operating conditions. Additionally, for ignition, an appreciable decrease is found for nAl particles in the range 0.1–0.2 μm, while little effect was observed for μAl. The minute nAl powder manifest a strong reactivity mainly due to their increased specific surface, notwithstanding the simultaneous decrease in active Al content (typically, from 99.6% of 17 μm Al to 88.2% for nAl produced by electrical explosion of wires) [98]. Within the current rocket propulsion technology, ultrafine particles, such as in the range 50–150 nm, are preferable to true nanosized particles. Maybe this will change in the future, but not in the short term [99].

Even if the 2P losses are mitigated, nanosized energetic ingredients with higher energy densities and faster energy release rates exhibited with respect to conventional ingredients arose great expectations in solid propulsion, but despite the intense worldwide investigation programs, use of nAl still today also entails several negative effects. Loss of active metal, clustering during manufacture and storage, EOM viscosity, possible impairment of mechanical properties, less effective acoustic damping, aging, and cost (even though it is nowadays much diminished) are more than enough to balance the expected advantages. Moreover, with increasing nAl fraction, friction sensitivity, impact sensitivity, but also the measured heat of combustion (because of higher combustion efficiency), all increase [96,99,100,101]. The recommended approach to get the best of both worlds is to resort to dual metallic fuels, properly blending μAl and nAl. Another approach to exploit the potential of nAl is particle coating, the complexities of which, however, require careful manufacturing and do not lead to immediate solutions [98]. Additionally, mostly laboratory level applications of metal nanopowders are reported and often for scientific purposes only. A number of practical reasons prevent the applications at industrial level, such as particles coating with inert materials, cost, aging, etc. A good control of particle size, metal content, and dispersion is a crucial requirement for applications of nanoingredients in propulsion.

## 6. Conclusions

Metal nanoparticles have wide application aspects in various explosives and propulsion systems, including advanced components of pyrotechnic compositions, effective burning rate modifiers of solid propellants, etc. Based on the investigations, the following conclusions can be drawn:

(1) Addition of nAl is beneficial to improve the burning rate and possibly reduce the burning rate pressure exponent, but at the same time, the low active Al content of nAl powder leads to a reduction the heat of explosion of propellants.
(2) A core–shell structure of nanoparticles can be obtained, and the burning rate of solid propellants can be increased using different material coatings on the surface of nAl. The addition of nTi, nZr can increase the density of propellant, while the heat of explosion of propellant decreases.
(3) Within the current rocket propulsion technology, mostly laboratory level applications of metal nanopowders are reported and often for scientific purposes; much work is needed for the applications of metal nanopowders at an industrial level.

## Figures and Tables

**Table 1:** Effect of nNi on the performance of CMDB propellants.

Samples	Methyl Violet, Change Color Time/Min	Vieri, Change Color Time/h	5 h Explode or Burn	5 s Outbreak Temperature/°C	Impact Sensitivity—*H*_50_/cm
RDX/nNi/others = 0/0/5.75	68	68.0	not	268.4	10.6
RDX/nNi/others = 0/0.7/5.05	69	68.5	not	258.0	13.7
RDX/nNi/others = 10/0.7/3.55	70	68.0	not	258.0	11.0
Samples	Friction sensitivity/%	Heat of explosion/J g^−1^	Specific volume/L kg^−1^	Density/g cm^−3^	
RDX/nNi/others = 0/0/5.75	85	4978	624	1.701	
RDX/nNi/others = 0/0.7/5.05	81	4963	626	1.701	
RDX/nNi/others = 10/0.7/3.55	72	4955	638	1.695	

**Table 2:** Summary of nano-metric metals and its effects on the performance of energetic compositions.

Types of Metals		Advantages and Disadvantages	Refs.
Carbon coating	nAl@C	A core–shell structure of nanoparticles was obtained.	[26,27]
Metals coating	nAl@B	The dispersion feature was improved, the onset oxygen temperature and the heat of combustion of Al were increased.	[28,29]
	nAl@Ni	The stability is good. The active Al content changes little after storage for one month in air. The burning rate of solid propellants increased.	[31,32]
Metal oxide coating	nAl@Al_2_O_3_	The agglomeration of particles reduced; the burning rate of solid propellants increases. The heat release behavior of nAl hindered, and the oxidation exothermic temperature increased. The active Al content reduced.	[34]
	nAl@CuO	with high reactivity and quick reactive speed.	[35,36,37,38]
Organic acid coating	nAl@OA	The exothermic peak temperature moved forward, the stability towards oxidation in air and in water increased.	[40,41,42]
	nAl@SA	The combustion performance is more complete, the stability towards oxidation in air and in water increased.	[43]
	nAl@PA	The active Al content decreased	[21,46]
	nAl@FS	The dispersity was improved, and the particle size distribution was more homogeneous. The ignition delay time is shorter, and the combustion reaction is more intense, the flame brightness is higher.	[47]
Polymer coating	nAl@GAP	The core–shell particles are observed, while the decomposition temperature increases.	[49,50]
	nAl@HTPB	The active Al content is less than 50%.	[51]
	nAl@NC	The stability to oxidation in air during the storage period increased, the reactivity by heating is high.	[52]
Energetic materials coating	nAl@RDX	The performance of Al in propellants improved; the activation energy reduced. The active Al content is up to 89%.	[53]
Fluoride coating	nAl@F	The active Al content is up to 85.85%, the combustion of nAl was promoted, and the heat of combustion is high. The regression rate of the fuel promoted.	[54,55,56]
	nAl@PF	A core–shell structure was observed. The energy amount and energy release rate is higher than that of nAl.	[58]
	nAl@Viton B	A core–shell structure was observed. The protective effect, the energy amount and energy release rate is higher than that of nAl.	[58]
	nAl@shellac	The energy amount and energy release rate are higher than that of nAl.	[58]
Other materials coating	nAl@AP	The ignition temperature is lower than that of nAl.	[59,60]
	nAl@DOS	The active Al content is low.	[61]
	nAl@PDA	The crystal form of Al remains unchanged before and after coating.	[62]
	nAl@NDZ	The active Al content and heat of explosive decreased. The burning rate of propellant increased, while the pressure exponent decreased.	[63]
	nAl@NGTC	The active Al content and heat of explosive decreased. The burning rate of propellant increased, while the pressure exponent decreased.	[63]
nNi		Addition of nNi to the propellant increases the burning rate and reduce the pressure exponent. Addition of nNi can slightly increase the mechanical sensitivity of propellant. The detonation heat, the heat of explosion and density of CMDB propellant decreased, while the specific volume increased.	[90,91,92]
nTi		The heat of explosion of propellant decreased, while the density increased.	[94,95,96]
nZr		The heat of explosion of propellant decreased, while the density increased.	[94,95,96]
nFe		The high temperature thermal decomposition peak temperature of AP is advanced, the propellant burning rate increases, while pressure exponent decreases.	[97]
nCo		Addition of nCo reduces the high-temperature decomposition peak of AP, the propellant burning rate increases, but the maximum activation energy and pressure exponent decreased.	[97]

## Data Availability

Not applicable.
